# Recurring Facial Erythema in an Infant

**DOI:** 10.1155/2016/9285496

**Published:** 2016-10-24

**Authors:** Sam Hassan, Mary Jacqueline Saviour

**Affiliations:** ^1^Department of Pediatrics and Neonates, Mediclinic City Hospital, Dubai Health Care City, P.O. Box 505004, Dubai, UAE; ^2^Mediclinic City Hospital, Dubai Health Care City, P.O. Box 505004, Dubai, UAE

## Abstract

Causes of facial rashes and erythema in infants are many but rarely only happen during feeding times which are commonly and sometimes wrongly attributed to food allergy. There is a rare condition called Auriculotemporal nerve syndrome that is characterized by recurrent episodes of gustatory facial flushing and sweating along the cutaneous distribution of Auriculotemporal nerve: the so-called Frey syndrome. This condition is most frequently observed in adults usually after parotid surgery. It is rare in children and is mostly attributed to forceps assisted delivery. It can also be misinterpreted as food allergy. Here we report a case of an infant with Frey syndrome without any history of perinatal trauma, which was considered initially as food allergy and highlights the importance of distinguishing it from food allergy.

## 1. Introduction

Frey syndrome is a common postoperative complication of parotid gland surgery in adults. It may also follow other surgical, traumatic, or inflammatory injuries of the parotid and submandibular gland [1]. Rarely Auriculotemporal nerve syndrome has been reported as a sequel of perinatal birth trauma resulting from assisted forceps delivery [2]. The presentation, diagnosis, pathophysiology, and treatment of pediatric Frey syndrome are reviewed here.

## 2. Case Report

A nine-month-old Romanian baby was brought to pediatric outpatient department with the history of multiple episodes of rashes on the right cheek since the age of 7 months. He was born in the same hospital by full-term, normal spontaneous vaginal delivery. The antenatal, prenatal, and postnatal history were normal. Baby was purely on breast feeding until the age of six months. Soon after the introduction of the first solid food right cheek became erythematous which resolved spontaneously after feeding has stopped. During this episode no rashes were seen on any other part of the body.

The rash was not associated with any itching or breathing difficulty or any other symptoms. But the rash reoccurred whenever the baby was given citrus fruits or vegetables, and it never occurred spontaneously; there was no associated sweating, rhinorrhea, lacrimation, or excessive salivation and no neurological abnormalities were noted.

The parents had a concern of excessive sweating of the body which resolved by itself. There was also a history of rashes appearing on the body while the baby was given bathing in the initial few months of life which resolved fully. There was no history of trauma or surgical operation apart from tongue tie release at the age of two months. There is no family history of any allergic disorders.

On clinical examination the baby was active and alert with normal systemic examination and achieved normal developmental mile stones in his age and normal growth parameters. Mild asymmetry was noted on the cheek. There were no lesions or abnormalities detected inside the oral cavity. The right cheek was found to be slightly firmer than the other.

There were no signs of atopic eczema, seborrheic dermatitis, or other skin diseases seen. There was no watering from eyes or nose, and there were not any wheezes or clinical signs of gastroenteritis symptoms.

In a previous visit to another clinic food allergy was considered as a diagnosis, a full-blood count and total IgE level were normal, and allergy tests for food panel came negative. In view of the mild asymmetry of the cheek an ultrasound of the soft tissue was performed which showed normal facial structures including the salivary glands and slight asymmetry of the subcutaneous fat which was more prominent on the right side. No underlying focal soft tissue lesion or abnormal vascularity was seen.

Parents were asked to video the case during feeding and it was also observed during the time of doing the US scan while feeding. It showed development of erythema on the right check extending to the right ear few minutes after starting solid food which was reported more with citric fruits and disappeared fully an hour after feeding ceased.

**Figure 1:**
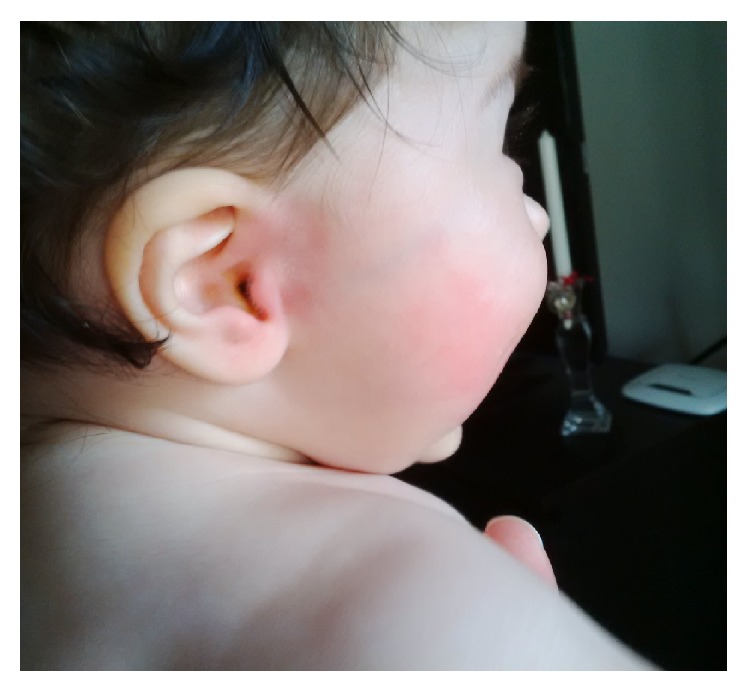
Erythema on the right cheek on eating citrus fruit.

The case was diagnosed as Frey's syndrome, upon the history, physical examination, and negative blood tests. Father provided us with the image which was taken at home while the event happened which is shown in Figure 1.

## 3. Discussion

Frey syndrome or Auriculotemporal syndrome was first described in 1757 by Duphex and named after Frey, a polish neurologist who identified the role of Auriculotemporal nerve in 1923 [3]. The syndrome is characterized by facial flushing, increased sweating, and warmth in the distribution of Auriculotemporal nerve, which occurs after taking acidic, sour, or spicy foods.

Auriculotemporal nerve is a branch of the mandibular nerve of the trigeminal nerve complex [4]. This flushing occurs from the corner of mouth and spreads diagonally across the cheek to the preauricular and temporal areas. Rarely this can also occur bilaterally.

Frey syndrome is also known as Baillarger's syndrome, Dupuy's syndrome, Auriculotemporal syndrome or Frey-Baillarger syndrome [5, 6]. This condition most commonly occurs as a squeal of parotidectomy. It may follow other surgical, traumatic, or inflammatory condition of the parotid gland.

**Figure 2:**
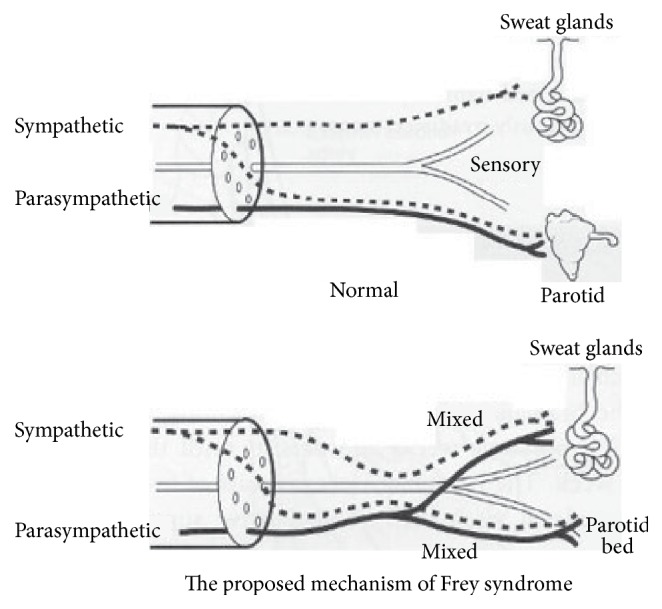
Pathophysiology [8].

The pathophysiology is not completely understood. Multiple theories have been proposed on the pathophysiology and the most accepted hypothesis is that trauma to the Auriculotemporal nerve leads to aberrant regeneration of the cut parasympathetic fibers between the otic ganglion and salivary gland tissue leading to innervation of sweat glands and subcutaneous vessels, as shown in Figure 2, which results in sweating and redness of the skin of the involved area following a gustatory stimulation [7].

Frey syndrome is less common in pediatric population and most of the cases occur during infancy. Even though an assisted forceps delivery has been attributed in pediatric cases, out of the 92 pediatric cases listed in the PubMed database from 1948 to 2015, 72 cases had no history of local surgery, postnatal trauma, tumor, or malformations [9].

Postulated mechanism for these cases without trauma or surgery include a congenital aberrant pathway, crossover of the fibers caused by loss of insulation around the neural sheaths, neural irritation due to scar tissue formation, subclinical intrauterine infection, and mild injury to the parotid gland [10].

Few cases are reported in pediatrics following preauricular lymphadenectomy and bicycle accidents resulting in mandibular condylar fracture [11–13].

Usually the skin changes seen in Frey's syndrome are initially observed around the time of introduction of first solid food particularly following ingestion of citrus fruits and skin erythema is the most common change and it usually lasts for 30–60 minutes and resolves spontaneously.

Unlike adults, children do not exhibit sweating over the affected area. This absence of sweating is not well understood but may be related to immaturity of exocrine glands in childhood and also to the less traumatic mechanism of the lesion [14].

The diagnosis of Frey syndrome is more challenging for pediatricians as this is often confused with food allergy. Allergic reaction accounts for majority of food associated skin reactions in children. This case was considered initially an allergic reaction to food, but the allergy test and other blood investigations were negative.

Allergic skin reaction could have been ruled out in the initial stage itself as the facial erythema was confined only to one side of the face and other systemic signs like itching, hives, breathing difficulty, or gastrointestinal symptoms were not present and the erythema happened also with different types of food.

Various methods have been developed in adults for diagnosing Frey's syndrome including minor starch iodine test and thermography and the use of questionnaire for the subjective assessment of symptoms.

Various forms of treatment including medical and surgical ones have been tried in adults. Intracutaneous injection of botulinum toxin is a safe and effective treatment in adults [15]. However, in children it is considered as a benign self-limiting condition. Long term follow-up has not been documented anywhere in the literature. As the child grows spontaneous reduction of symptoms occur and, for the same reason, no treatment is required. Haxton described a child with Frey's syndrome who underwent cervical sympathectomy and subsequently developed Horner's syndrome [16, 17].

## 4. Conclusion

Frey syndrome is a rare phenomenon in pediatrics. Awareness of this condition and its recognition are important mainly for the pediatricians and primary care physicians to avoid unnecessary referrals, costly investigations, and dietary restrictions. The diagnosis is always based on appropriate history taking and careful observation of the patient during feeding. Early clinical diagnosis and reassurance of the parents avoid multiple outpatient consultation and reduce parent anxiety.
